# Case of Ovarian Dropsy, Successfully Removed by a Surgical Operation

**Published:** 1822-04

**Authors:** Nathan Smith

**Affiliations:** Professor of Physic and Surgery at Yale College.


Art. VII.—*Case of Ovarian Dropsy, successfully removed by a Surgical Operation.* Communicated by Dr. Nathan Smith, Professor of Physic and Surgery at Yale College. (From the American Medical Recorder, No. 17.)

THE subject of this operation was a Mrs. Strobridge, of Norwich, Vermont, aged thirty-three years.

The following account of the case, previous to the operation, was taken from the patient:—Seven years before, she perceived a small tumor in her right side, situated in the right iliac region: when about the size of a goose-egg, she could move it with her hand to the opposite side of the linea alba, and to some distance above the umbilicus. The patient had borne five children, two previous, and three subsequent, to her discovering the tumor.

The youngest child was ten months old, and was nursed at the breast when she submitted to the operation. Soon after her first pregnancy from the commencement of the tumor, and when, as she thinks, it was about four or five inches in diameter, it suddenly disappeared,—probably burst into the abdomen. In four or five weeks it was as large as before. Before and after the bursting of the tumor, she had turns of faintness, which lasted from two hours to half a day. During parturition of her second child, after the commencement of the tumor, it having acquired a considerable size, it burst again; and nothing was perceived of it till eight months had elapsed. In four days from its re-appearance, it was as large as it had ever been. It was again burst by a fall: great soreness of the abdomen, and confinement of the patient for several weeks, was the consequence. The tumor filled again in a fortnight, and from this time continued to increase: it did not burst in the delivery of her last child, which was ten months previous to the operation. The patient's health was not much affected by the tumor. She was costive; and the size of the tumor incommoded her in the ordinary duties of her family, especially in stooping. On examination, I found a large tumor in the right side of the abdomen: it was considerably moveable, and I could perceive a distinct fluctuation through it.

Having decided on the operation, and determined the mode of operating, on the 5th of July, in the presence, and with the assistance, of Doctors Lewis, Mussy, Dana, and Hatch, I commenced the operation, as follows:

The patient being placed on a bed, with her head and shoulders somewhat raised, an assistant rolled up the tumor to the middle of the abdomen, and held it there. I then commenced an incision about an inch below the umbilicus, directly in the linea alba, and extended it downwards three inches. I carried it down to the peritoneum, and then stopped till the blood ceased to flow, which it soon did. I then divided the peritoneum the whole extent of the external incision. The tumor, now exposed to view, was punctured; a canula introduced, and seven pints of a dark-coloured ropy fluid was discharged into a vessel: about one pint was spilt, so that the whole fluid was about eight pounds. Previous to tapping the tumor, by inserting my finger by the side of it, I ascertained that it adhered, to some extent, to the parietes of the abdomen on the right side, between the spine of the ileum and false ribs. After evacuating the fluid, I drew out the sac, which brought out with it, and adhering to it, a considerable portion of the omentum. This was separated from the sac with the knife; and two arteries, which we feared might bleed, were tied with leather ligatures, nd the omentum was returned. By continuing to pull out the sac, the ovarian ligament was brought out: this was cut off, two small arteries secured with leather ligatures, and the ligament was then returned. I then endeavoured to separate the sac from its adhesions to the parietes of the abdomen, which occupied a space about two inches square: this was effected by a slight stroke of the knife at the anterior part of the adhesion, and by use of the fingers. The sac then came out whole, excepting where the puncture was made, and I should think it might weigh between two and four ounces. The incision was then closed with adhesive plaster, and a bandage applied over the abdomen. No unfavourable symptoms occurred after the operation: in three weeks the patient was able to sit up and walk, and has since perfectly recovered.

I was induced to undertake this operation from the following considerations:—The patient, though her health was not greatly impaired, was sensibly affected by the disease. She was quite certain that the increase of the tumor, in a given time, was augmented: probably, at no very distant period, it would destroy her. I had, also, an opportunity to dissect the body of a patient, who had died of ovarian dropsy after being tapped seven times. In this case the sac was found to be in the right ovarium, which filled the whole abdomen; but it adhered to no part except the proper ligament, which was not larger than the finger of a man. I have seen two other ovarian sacs which were taken from patients after death. They had been tapped several times; the sacs were equally unattached, except to their own proper ligaments. Hence I inferred, that, in a case of ovarian dropsy, while the tumor remained moveable, it might be removed with a prospect of success. The mode of operating practised in the above case, is the same as I have described to my pupils in several of my last courses of lectures on surgery. The event has justified my previous opinions.

